# Nutritional Intake and Sensory Processing in School-Aged Children with Autism Spectrum Disorder

**DOI:** 10.3390/nu17040604

**Published:** 2025-02-07

**Authors:** Audrey Olson, Jenna R. Krall, Ancha Baranova, Margaret Slavin

**Affiliations:** 1 School of Systems Biology, George Mason University, Manassas, VA 20110, USA; aolson2@gmu.edu (A.O.); abaranov@gmu.edu (A.B.); 2 Department of Global and Community Health, George Mason University, Fairfax, VA 22030, USA; jkrall@gmu.edu; 3 Department of Nutrition and Food Studies, George Mason University, Fairfax, VA 22030, USA; 4 Department of Nutrition and Food Science, University of Maryland, College Park, MD 20742, USA

**Keywords:** B vitamins, folate, zinc, choline, autism, sensory processing, sensory dysregulation, one-carbon metabolism, sensory profile 2, YAFFQ

## Abstract

**Background:** Individuals diagnosed with autism spectrum disorder (ASD) commonly experience sensory processing that differs from general-population norms, and the autistic lived experience of eating includes preferences for routine, and sensory processing difficulty related to scents, tastes, temperatures, and textures of food. Meanwhile, research indicates that nutrients involved in one-carbon metabolism (OCM) may be related to sensory processing. **Methods:** This study enrolled 33 school-aged children with autism to assess whether OCM nutrient intake is associated with sensory processing. Parents completed two parent-report assessments: the youth and adult food frequency questionnaire (YAFFQ), and a sensory processing tool, Sensory Profile 2 (SP2). **Results:** Participant data showed generally good nutritional profiles mirroring those of general-population U.S. children. A group-binarized linear regression model showed the following relationships (*p* < 0.05): vitamin B12 consumption had a negative association with the SP2 Oral and Sensor domain scores. Choline intake had a positive association with the SP2 Avoider domain score. Vitamin B1 showed a positive association with the SP2 Visual domain score. **Conclusions:** These results support the possible existence of a relationship between sensory symptoms and OCM nutrient consumption levels in school-aged children diagnosed with autism. Future research is needed to confirm and explore the potential for causality.

## 1. Introduction

Autistic people inherently intersect with food and nutrition in a unique way. Autistic individuals—and particularly autistic children—often express high degrees of food selectivity, meaning that autistic adults and children alike may accept a very limited range of foods, refuse foods, or be unwilling to try new foods. Sensory hyper- or hyposensitivies and their related behaviors are core symptoms of autism spectrum disorder (ASD, also written here as autism), recognized in the diagnostic criteria in the DSM-V and experienced by 69–95% of the autistic population across their lifespan [1,2]. These sensory processing challenges are individualized and occur across a wide range of domains, including visual, auditory, touch, oral, and vestibular domains [3,4,5,6,7,8,9,10,11,12,13,14,15,16,17]. One example of sensory processing differences across these domains in autism can be seen in sensory gating, which describes the habituation of a person to sensory inputs based upon how often neuron signals communicate sensory information to the brain [18,19,20]. This function allows human brains to register which sensory input is new, stronger, or salient, and which is redundant, and therefore, ignorable, protecting the brain from having to process already-known information about its environment. Sensory gating that is less strict allows sensory signals to be registered more easily, more frequently, and without habituation. Sensory gating that is stricter requires stronger, different, and/or more persistent sensory signals before they are communicated to the brain. In principle, sensory gating can apply to all sensory processing domains, including oral hypersensitivity, which has particularly been identified as a significant risk factor for food selectivity and limited dietary variety in children with autism—but other research suggests that this may not de facto lead to nutrient deficiencies [21,22].

**Figure 1:**
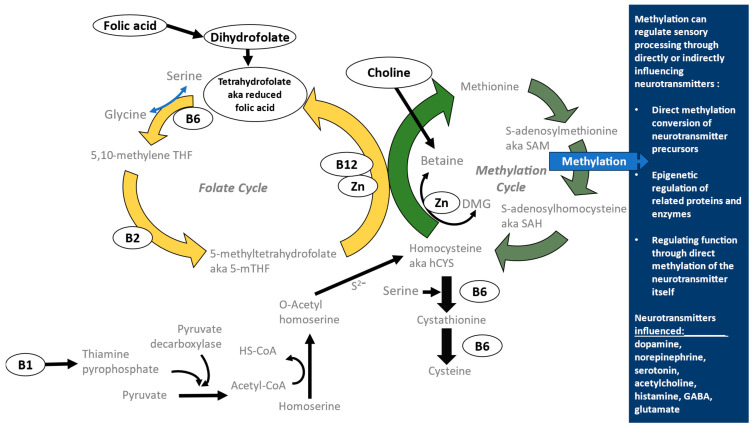
One-carbon metabolism nutrients and related pathways. One-carbon metabolism (OCM) relies heavily upon B vitamins, folic acid, and zinc. Often, B vitamins serve as cofactors in conversion processes, both within OCM and just upstream of it, as in the case with vitamin B1 assisting in the conversion of pyruvate to acetyl-CoA, which ultimately helps to supply homocysteine for the methylation cycle. Insufficient consumption, and, eventually, the deficiency of these nutrients could hinder OCM and the production and regulation of neurotransmitters conducting sensory processing throughout the human body.

One-carbon metabolism (OCM) is a fundamental biochemical process permitting the usage of single-carbon units in vital processes such as DNA and RNA synthesis, protein production, and gene-expression-regulating methylation. A growing body of evidence suggests that nutritional intake, and particularly the intake of certain nutrients linked to one-carbon metabolism (Vitamins B1, B2, B6, folate, B12, choline and zinc, hereafter referred to as ‘OCM nutrients’; see Figure 1), may influence sensory signal pathways, sensorimotor response, and ultimately, sensory symptoms [23,24]. OCM is essential for the production of sensory-processing-dependent neurotransmitters, such as serotonin, dopamine, and norepinephrine, and OCM metabolites are measurably different in autistic children compared to their neurotypical peers [25]. In particular, choline has been linked to sensory processing and related behaviors in a number of ways: animal choline deficiency models in utero [26] and in vivo [27] produce sensory-processing impairment. Conversely, choline supplementation showed improvements in auditory gating in a subgroup of human adults with low (i.e., suppressed) auditory gating [28], in social interaction deficits in the offspring of a mouse model of autism [29], and in reducing repetitive behavioral symptoms in adult offspring of MTHFR+/− mouse mothers [30]. Mechanistic modeling of metabolism pathways also supports that a relationship between dietary choline and sensory symptoms is plausible [31].

Meanwhile, B vitamins are mechanistically interrelated with choline in their participation in distinct aspects of one-carbon metabolism, such as the cycle converting homocysteine to methionine, and its interplay with the folate cycle’s related methyl group transfer. Folic acid (B9) supplementation in children with autism improved core symptoms of autism, as well as improved concentrations of homocysteine [32]. Folate intake is associated with one-carbon metabolites in children diagnosed with autism, and in certain behaviors that are tied with sensory processing symptoms in the literature [33]. In an environmentally induced animal model of autism, gestational administration of B vitamins was protective against synaptic loss and neurobehavioral impairment in offspring, and produced reductions in repetitive behaviors often associated with sensory symptoms [34]. Separately, supplementation of Vitamins B6 and B12 plus magnesium in children with autism stabilized urinary tryptophan excretion [35]. Tryptophan is of interest because it is a precursor to serotonin and other neurotransmitters, and serotonergic differences have been associated with autism and its symptoms. Collectively, these results show the potential for OCM nutrients to modulate sensory symptoms associated with autism.

Given these complexities, if the option to mitigate sensory symptoms via nutrition exists, it would serve as a plausible avenue for improving the quality of life of autistic people. Further, such an option aligns with one of the stated priorities of the autistic community: to improve the management of sensory symptoms [36].

As one means of exploring the relationship between OCM nutrients and sensory symptoms associated with autism, this study aimed to observe nutritional intake in children ages 6 to 10 years with a diagnosis of ASD, and to further assess whether dietary intake of OCM nutrients was associated with sensory processing outcomes.

## 2. Methods

### 2.1. Study Population and Recruitment

Families of children aged 6–10 years with a professional diagnosis of autism spectrum disorder were recruited from Northern Virginia. Inclusion criteria also required participants to be residing within the U.S. Exclusion criteria were other medical diagnoses which would impact nutrient metabolism (such as inborn errors of metabolism), and the use of medications which impact the metabolism of choline. If using choline supplementation, the regimen must have been stable for at least 6 months for a participant to qualify to join the study.

Recruitment was conducted through sharing flyers with local autism advocacy groups, therapy clinics, and parent–teacher organizations, and at in-person advocacy events (i.e., fundraising walks). Between June 2019 and March 2020, parents completed an initial screening phone call with researchers and were subsequently invited to an in-person meeting to complete the consent process and the initial questionnaires. Recruitment was paused due to the coronavirus pandemic between mid-March 2020 and October 2020, at which point recruitment, screening, consent, and data collection were moved fully online, with recruitment conducted through social media and advocacy group distribution lists, and data collection conducted via REDCap questionnaires online. Parents completed all questionnaires on behalf of their child. All versions of the protocol were approved by the George Mason University Institutional Review Board under project #1357626.

### 2.2. Demographics, Health and Diet History

A brief demographic, health, and diet history questionnaire was collected from parents, in paper form and electronically via REDCap, during the pre-COVID-19 and COVID-19 periods, respectively. In addition to the collection of basic demographic data, parents were asked to list other medical diagnoses, current regular medications and dietary supplements, and other treatments for ASD that the child has received in the past 12 months, and specific diets that the child has ever followed. Parents were also asked to describe their child’s food preferences in an open-ended question. The open answers to the child’s food preferences were coded for indications of food selectivity by two researchers (AO, MS): children whose parents used words like ‘picky’, ‘very picky’, ‘(very) limited diet’, ‘selective’, and descriptions of sensitivity to texture were coded as being food-selective.

### 2.3. Dietary Assessment

Dietary data were collected via the Harvard Youth Adolescent Food Frequency Questionnaire (YAFFQ). The YAFFQ is a 126-item quantitative FFQ which estimates daily nutrient intake based upon consumption patterns in the child’s dietary intake and supplement use over the past 12 months. It has been validated for older children and adolescents (aged 9 years and up) for self-administration [37] and has been used in younger populations with parental assistance and parental completion in children with autism who are within ages of 3–11 years [38]. Parents completed the YAFFQ on behalf of their child. For analysis, variables for total intake (diet + supplements) are presented.

### 2.4. Sensory Assessment

Parents were asked to complete the Child Sensory Profile 2 (SP2) [39,40,41] questionnaire (Pearson Clinical) about their child. The SP2 is an 86-item questionnaire which asks caregivers to rate the child’s responsivity and frequency of behaviors in response to sensory events; it is intended for use in children between the ages of 3 and 14 years, which quantifies scores across 13 sensory-processing domains [42]. It is commonly used in occupational therapy settings with demonstrated content and convergent validity [39]. The 2014 SP2 is a population-norm-referenced means of assessment that attempts to address shortcomings identified in two assessment tools that preceded it in clinical use: the Short Sensory Profile (SSP, 2002) and the Sensory Profile (SP, 1999) [39,42,43,44].

### 2.5. Statistical Analysis

Age, ethnicity, and gender were described with descriptive statistics, and medications and supplements were categorized before counting. To account for the skewing of the nutrient intake data, the intake of each of the 7 OCM nutrients (Vitamins B1, B2, B6, B12, folate, choline, and zinc) was dichotomized using the median, creating a binary variable representing each individual nutrient’s intake as either below or equal to/above the dataset median for each nutrient, respectively.

This created a dataset with a binarized nutrient status for each nutrient as the independent variable, and the raw sensory processing scores, respectively, by domain, as the dependent variable. Taking this study’s small sample size and exploratory nature into account, as well as the mixed data types, a linear regression model was chosen. This reflects the literature that acknowledges linear regression with sample sizes as small as 30 as valid, particularly with binary predictors and continuous outcomes [45,46,47]. Potential adjustment variables were identified based on the biological plausibility of impacting both nutrient metabolism and sensory symptoms through the literature review and a sensitivity analysis—identifying age, the use of ADHD medication, and caffeine intake as adjustment variables that were included in the final model.

## 3. Results

### 3.1. Participants

**Figure 2:**
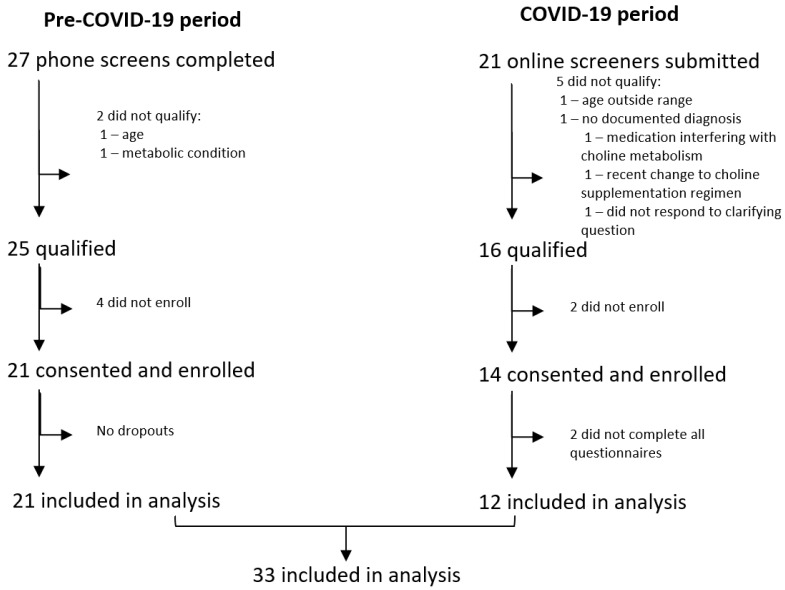
Participant flowchart.

Thirty-five participants enrolled in the study after passing an initial screening. Thirty-three (33) participants completed all questionnaires and were included in analyses, while the remaining two did not complete one or more questionnaires. Of the 33 participants, 21 were enrolled in-person pre-pandemic and 12 participants enrolled online during the pandemic period (See Figure 2).

Demographic characteristics of the participants are presented in Table 1. In addition, the most commonly reported co-diagnosis was attention deficit hyperactivity disorder (ADHD, *n* = 15), followed by anxiety (*n* = 4). Parents reported that five children had food allergies or intolerances. A total of six children were following a special diet, typically by excluding some combination of foods (of these six children, five were following a gluten-free diet, four were following a dairy- or casein-free diet, four were avoiding sugars, one was avoiding soy, and one was avoiding Red40 food dye), in addition to avoiding peanut (*n* = 2) and tree nut (*n* = 1) allergens.

### 3.2. Dietary Intake

Table 2 describes the mean intake for including OCM nutrients: choline, zinc, and Vitamins B1, B2, B6, folate and B12. The percentage of study participants consuming sufficient amounts, according to standard recommendations according to their age/gender (i.e., Dietary Reference Intakes or Dietary Guidelines for Americans), is also presented. The expanded version of this table—Supplementary Table S1—describes in full the mean energy, macronutrient, and micronutrient intakes of participants.

### 3.3. Sensory Analysis

The results of the sensory analysis questionnaire are presented in Table 3, presented as SP2 raw scores. The mean scores for all four quadrants of the sensory profile (Seeking/Seeker, Avoiding/Avoider, Sensitivity/Sensor, Registration/Bystander) registered in the “more than others” category according to the SP2 report ranges. Table 4 shows the overall participant tallies by SP2 classification.

### 3.4. Micronutrient Intake and Sensory Symptoms

The generalized linear model examined potential relationships between median-binarized OCM nutrient intake, with binarized intake as the explanatory variable of interest, adjusted for age, ADHD medication status, and caffeine intake, and sensory processing scores as the outcome (Table 5). This model identified an association between the intake of nutrients and sensory processing scores in four cases (*p* < 0.05). For choline intake, there is a positive association with avoider scores (*p* = 0.044). For vitamin B12, there is a negative association with the sensor scores (*p* = 0.046). For visual sensory scores, a positive association was found with the intake of vitamin B1 (*p* = 0.026). Finally, for oral sensory scores, a negative association was found for vitamin B12 (*p* = 0.026).

A subsequent generalized linear model (Supplementary Table S3) assessed a potential relationship between oral sensory processing raw scores and intakes of OCM nutrients below DRI-recommended levels of intake using the same adjustment variables of age, caffeine intake, and ADHD medication status. No statistically significant relationship was observed between oral sensory processing scores and a below-recommended DRI of choline, Vitamins B1, B2, B6, B12, folate, or zinc.

## 4. Discussion

### 4.1. Study Participant Nutritional Intake and the Extant Literature on General Nutritional Status in Autism

The nutritional intake of study participants, combining food and supplement intakes, was largely adequate as assessed by the YAFFQ. The majority of this study’s participants met the DRI recommendation for most nutrients (Table 2, Supplementary Table S1). For example, over 80% of participants met their respective DRI for vitamin B1, vitamin B2, vitamin B5 (pantothenic acid), vitamin B6, folate, vitamin B12, vitamin C, iron, zinc, and magnesium. While many nutritional studies of autistic children have observed restricted dietary intake patterns and warn of general malnutrition in autistic children across a variety of nutrient categories [48,49,50,51,52], there are other studies which generally agree with the results of the present study in that intakes are largely sufficient, and further, that children on the autism spectrum are performing about as well as their peers in terms of micronutrient consumption and are largely without risk of deficiency [25,53]. This runs counter to the much earlier literature on nutrition in autism, which once claimed that about half of autistic children may have nutrient deficiencies [54].

However, the achievement of the DRI for several micronutrients was still low in this study, where about one third to one half of participants had consumption below the DRI, including choline, potassium, calcium, and Vitamin D, with 36%, 54%, 52%, and 39% meeting their DRI, respectively. The low rate of achieving the choline DRI for the autistic children in this study is consistent with choline assessed in other studies, both in autistic children specifically [55] and children within this age group nationally [56], which once more aligns with the growing record of published evidence indicating that autistic children consume many nutrients at about the same level as their peers [25,53]. Choline was also the only OCM nutrient of which intake was below the DRI. Regarding low amounts of calcium intake, others have observed a similar pattern of intake in this subpopulation, where the mean daily intake of calcium appears to be near the DRI, but disparities in consumption levels mean that a large portion of the population is well below the DRI, while others consume much greater than the DRI [57]. Hyman et al. (2012) also observed a low intake of potassium and Vitamin D in a similar population of school-aged autistic children, along with low intakes of fiber, choline, and calcium, findings which match those observed in this study; Hyman’s work also reports similar trends in age-matched controls without autism [53]. Meanwhile, sodium intake is observed to be high in comparison to the CDRR, and is aligned with the sodium intake of this age group, generally [58].

In macronutrient terms, the overall balance of carbohydrates, fats, and proteins was in line within recommended proportions. However, the intake of added sugar and saturated fat at 11.6% and 10.6% of energy intake were both above the recommended 10% limit of calories for each, yet these numbers were again aligned with similar observations of overconsumption of added sugar and solid fat intakes in all children of this age group in the U.S. Dietary Guidelines report [59]. Fiber intake accounted for an average of 11.5 g/1000 kcal consumed, which is once more simultaneously lower than the 14 g/1000 kcal recommendation from the DRIs while also being aligned with commonly observed fiber underconsumption in this and other age groups.

None of the children were reported to have received nutritional counseling or dietary therapy within the past year, which is surprising in the context that 1 in 5 were following a specific diet, 2/3 were taking at least one dietary supplement, and 2/3 displayed some level of food selectivity (Table 1). This highlights an opportunity for greater involvement of nutrition professionals at present in supporting nutritional adequacy and the quality of life within the children’s food preferences and dietary restrictions. If future research connects OCM nutrient intake to improvements in sensory processing, this would warrant further involvement of nutritional professionals in the care of autistic individuals.

### 4.2. Nutrient Intake and Sensory Processing

This study aimed to look beyond nutritional adequacy as determined by standard definitions to observe if OCM nutrient intake levels could be related to sensory symptoms, as assessed through SP2 ratings.

#### 4.2.1. General Consistency with the Current Literature on Sensory Processing: Differences in Intake vs. Deficiency

The observed relationship between oral sensory processing and median-binarized vitamin B12 intake is generally consistent with the extant literature focusing on sensory processing and intake levels of food groups and micronutrients. It is critical to identify whether other studies show an association between sensory processing outcomes and *insufficient or deficient* (vs. simply lower) consumption of particular nutrients.In examining other published research, there are two directly related assessment tools whose outcomes may be reasonably compared with those of the 2014 SP2—the sensory processing assessment tool used in this study. Although the 2014 SP2 is the successor to the two assessment tools that preceded it in clinical use, the Short Sensory Profile (SSP, 2002) and the Sensory Profile (SP, 1999) [39,42,43,44], in recent years, all three of these sensory assessment tools have been used in part or in whole to identify similar relationships between sensory processing symptoms and food/nutrient intake patterns [43]. Crucially, all three tools descend from the same 1997 sensory processing framework in studies of autistic children, which makes the rough comparison of outcomes measured by any of these three assessments reasonable.

Although oral sensory processing difficulties have been observed as related to decreased vegetable intake, this does not automatically point to nutritional deficiency [21]. Shmaya et al. tracked sensory processing in autistic children using the Sensory Profile, and found outlier sensory processing scores to be associated with formal assessment identification of mealtime behavior difficulties, but *not* nutritional deficiencies. In the same study, meal-time behavior difficulties were also not linked to nutritional deficiencies [22]. Similarly, our study’s DRI-defined deficiency status with respect to raw oral sensory processing scores did not show a statistically significant relationship between these scores and the deficiency status of any of the OCM nutrients. Even where a statistically significant negative relationship between oral processing scores and the group binarized intake of B12 is identified, higher oral sensory processing scores (above the population norm) within this group of 33 participants are *not* linked with deficient vitamin B12 intake—nor with the deficient intake of any other of these OCM nutrients (Supplementary Table S3).

#### 4.2.2. Consistency with the Literature on SP2 Scoring in Autism

**Figure 3:**
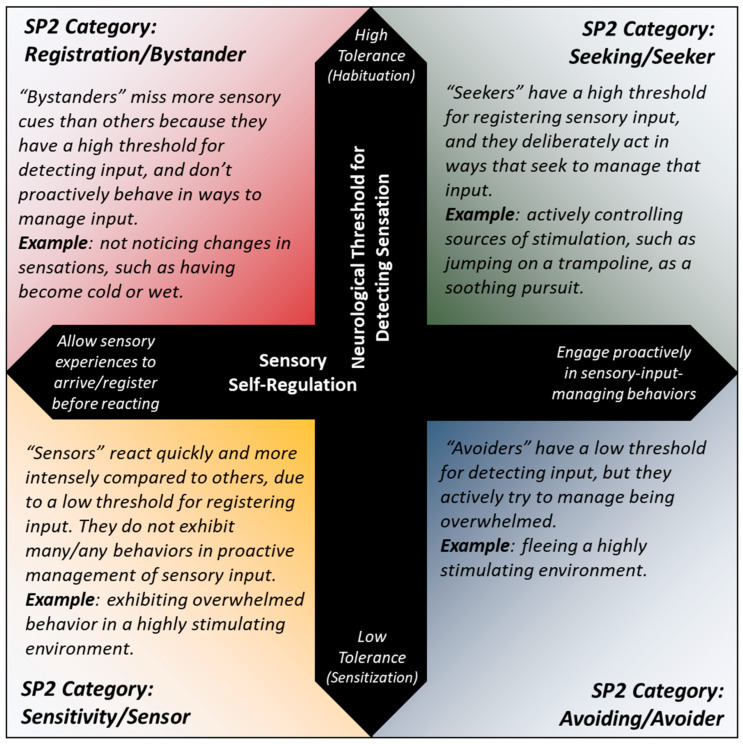
Quadrant characterizations for Sensory Profile 2. This illustration is adapted from the proposed quadrant framework by Dunn in 1997 [39,43]. According to Dunn, sensory processing behaviors can be contextualized when fitted along two continua—one for the neurological threshold for registering sensation, and another for the degree to which a child proactively manages their own sensory inputs (whether consciously or unconsciously). The quadrants formed by these continua are correspondingly defined as four distinct sensory-processing domains of behavior on the SP2.

The SP2’s Seeker, Avoider, Sensor, and Bystander domains are four domains which are interrelated, in terms of how an assessed child might be positioned on two continua: one for the neurological threshold at which the child registers sensory input, and the other for how proactively the child self-manages sensory inputs—referred to as self-regulation. The scoring patterns for this study’s children within these four domains were similar to those described in a detailed study of sensory quadrant scoring patterns in autistic children and adults, compared to their neurotypical peers [60,61]. This study’s characterization of quadrant patterns, which used the Sensory Profile, built upon Dunn’s framework of sensory processing—the theory upon which the SP2 assessment and its predecessors like the Sensory Profile were designed [39,43,62,63,64,65] (Figure 3).

Other research supports that the general sensory scoring profile in this study’s group of children across the other nine domains—where scores mostly trend as higher than the mean in a general pattern of behaviors signaling sensory over-responsiveness—is consistent with the previous literature, including a review assessing sensory processing behaviors in autism vs. control groups, which included many studies’ worth of Sensory Profile scores [39,44,60,61,64,65]. The review by Ben-Sasson et al. [60], particularly, showed generally large positive effect sizes for autism group sensory processing scores (compared to controls) related to scoring for under-responsiveness behaviors, over-responsiveness behaviors, and sensory-seeking behaviors.

#### 4.2.3. Vitamin B12 Consumption with Respect to Oral Sensory Processing

This study’s median-binarized statistical model shows a relationship between vitamin B12 consumption with respect to oral sensory processing scores, with the exception of choline. Because the oral sensory processing relationship to vitamin B12 intake is a negative association, this implies that B12 intake above the participant median is associated with generally lower oral sensory processing scores (in the range of 5.9–7.8 points lower for those consuming above the median intake). That is, those consuming an above-median intake of B12 were observed by their parents to exhibit fewer behaviors related to oral sensory processing differences. In the literature, this is sometimes inferred as a case of oral sensory sensitivity causing insufficient intake of vitamin B12 [66]. However, this does not rule out that one carbon metabolism - supported by adequate B12 intake - may sufficiently regulate neurotransmitter production to impact oral sensory processing behaviors. From this cross-sectional data, it cannot be determined whether this reflects lower B12 intake being partly responsible for higher oral sensory processing scores, or higher oral sensory processing scores influencing the mealtime intake of these OCM nutrients, or both. Further, because SP2 scores can exist at extremes on either side of the general population mean, there is never a clinical application default to lower or raise sensory avoidance scores.

#### 4.2.4. Choline and Sensory Avoidance Domain Response; Vitamin B12 and Sensor Domain Response

This study observes a positive association between median-binarized choline intake and sensory avoidance scores—which is to say, children consuming choline above the study median were observed as having higher sensory avoidance scores. Meanwhile, median-binarized vitamin B12 intake is positively associated with Sensor domain scores, indicating that children consuming vitamin B12 in amounts above the study median were observed as having higher Sensor domain scores. This means that children consuming above the median amounts of these nutrients, respectively, were likelier to have a higher score for the associated domain, implying increased amounts of behaviors defined as Avoider domain behaviors (for choline) and Sensor domain behaviors (for B12). (See Figure 3 for the SP2 characterization of these behaviors.)

Greater incidence of Avoider domain behaviors, according to the continua in Figure 3, implies that those eating above the choline median amount may have a lower neurological threshold for detecting sensory stimuli. This runs contrary to the current evidence base for choline as it directly relates to quantitative measures of sensory processing regulation. One 2014 study indicated that significant CDP-choline supplementation in a double-blind trial of healthy volunteers improved sensory gating and suppressed the response to the second stimulus in a rapidly delivered double dose of auditory stimuli (pre-pulse inhibition testing)—implying that the listener’s sensitivity to the second signal was dulled in a functional habituation to the audio source [28]. This effect of choline regulating sensory inhibition (the filtering of repetitive stimuli) through comparison with choline deficiency during gestation was also seen in rat model research [26].

Greater incidence of Sensor domain behaviors similarly implies a lower threshold for registration for those eating above the group median amount of vitamin B12. This is somewhat consistent with the literature on vitamin B12 consumption. For example, one study showed a 25% larger local field potential—triggered through whisker stimulation—measured in rats who had vitamin B12 supplementation. The authors noted, “We suggest that this enhancement might be the result of lowered sensory threshold, although the underlying mechanism has yet to be elucidated” [67].

Both of these domains—Avoider and Sensor—are specific to lower neurological thresholds for detecting sensory input. This hints that future research may stand to particularly clarify relationships between OCM nutrient intake and behaviors related to low sensory threshold activation. Until then, the directionality of these associations, if any, cannot be ascertained with current data. This group’s scores trend higher than those of the population score means. However, Sensor domain scores and Avoider domain scores for any one individual may exist on either extreme of general population means. Therefore, clinically, there is no default goal to raise or lower scores; it depends upon the individual’s scores and goals for sensory processing management.

### 4.3. Directions for Future Research

#### 4.3.1. Randomized Controlled Trial(s) as Key to Identifying Cause Directionality

Rigorous controlled lines of inquiry with washout periods could examine the direction of this study’s identified relationships more fully. To assess any relationship between an OCM nutrient’s intake and SP2 scores, the most thorough method for future research would be a double-blind crossover, randomized, controlled trial with a placebo, and intermediate washout timeframes. The careful study design detailed below could help to fortify the quality of the data and the related interpretation.

#### 4.3.2. Suggestions for Future Trial Research

##### Inclusion of Autistic Stakeholders in Future Research Design from Planning Stages

The future research proposed here requires the scrutiny and endorsement of autistic people as stakeholders. Autistic people describe improving their quality of life as one of their central goals for autism research [68,69,70,71]. It is widely recognized that food can influence quality of life through culinary or cultural influences. For autistic people, dietary subcultures exist with unique interactions of food and quality of life. One example is autistic people’s deep enjoyment of and reliance upon “samefoods”, that is, personalized subsets of foods often serving as the core of an autistic person’s diet, that can offer comfort or delight, particularly due to their dependable taste, smell, temperature, or texture [72,73,74,75,76]. In terms of trial design, autistic stakeholders can offer insight as to whether prospective food changes (and their resulting sensory changes) truly hold a high enough value to autistic people themselves to merit a dramatic dietary shift [77,78,79], or whether nutritional supplement formats (pills, powders, drops) would be preferable to changes to preferred routine dietary intakes.

##### Prioritize Examining OCM Nutrient Intake as the Independent Variable

When examining the two variables from this study—SP2 scores vs. OCM nutrient intake—OCM nutrient consumption emerges readily as the more controllable of the two, and is the obvious candidate to be the independent variable in a controlled trial. In contrast to dietary intake from foods, OCM nutrient intake control may be achieved most simply through nutritional supplementation, and relatedly, such a format allows for the administration of a placebo to enable proper blinding in a controlled trial.

##### Careful Research Timeline Selection

Sensory processing, and SP2 scores specifically, are impacted over time simply from any given child’s personal arc of development. Therefore, tight timelines are essential to prevent natural development arcs from clouding results. Related to timeframe duration choice, any trial should also ideally not overlap with a period of intense demand (such as common holidays) or transition (such as the beginning of the school year).

##### Enhance Trial Quality with Careful Controls, Additional Assessment Tools, and the Involvement of Sensory Processing Experts

The risk of bias resulting from repeated SP2 assessments in such a controlled trial can be mitigated in two ways. First, the placebo aspect is crucial, as it may help to identify to which extent SP2 scores change when parents, as observers, believe their child may be receiving a nutritional supplement meant to change their sensory behaviors. Second, the inclusion of sensory processing data from other sensory processing assessment tools conducted by professionals would constitute a high-quality validation to parent-report SP2s, and would enhance overall data quality. Qualified professionals, such as licensed, registered occupational therapists (OTR/Ls), should assess children while being blinded to their intervention status (classic control, placebo, or recipient of an OCM nutrient supplement).

#### 4.3.3. Clinical Applications’ Development After Future Controlled Trials

If future controlled research on OCM intake and sensory symptoms identifies a meaningful impact in either direction, adapting clinical practices based on this future research will require intensive collaboration and coordination among researchers, autistic stakeholders, and clinical professionals in related but distinct fields: registered dietitian nutritionists (RDNs), OTR/L therapists, speech language pathologists (SLPs), and other medical professionals such as gastroenterologists. All professionals involved would require a strong understanding of the SP2 scoring distribution and individual client goals [39,80].

### 4.4. Strengths and Limitations

A strength of this study is that its demographics were fairly well-matched relative to the area of recruitment (Northern Virginia), despite its modest sample size of 33 participants. This study’s participant demographics were 57.6% White, non-Hispanic (*n* = 19); 6.1% White, Hispanic (*n* = 2); 12.1% Black (*n* = 4); 12.1% Asian (*n* = 4); and 12.1% biracial (*n* = 4). When comparing these to demographics in Northern Virginia (NOVA) as a region, specifically, the area which the all of this study’s participants came from (20 Virginia county or city jurisdictions per the 2020 US census), the demographics of this participant group also align roughly with the NOVA population at 51.2% White, 17.4% Hispanic, 16.3% Asian, 14.1% Black, and 2.4% Other [81]. Additionally, the condensed format of the study, whose data collection required a single morning of assessments from a parent (child not required to be present) may have served as a strength in that it enabled more families to participate.

**Figure 4:**
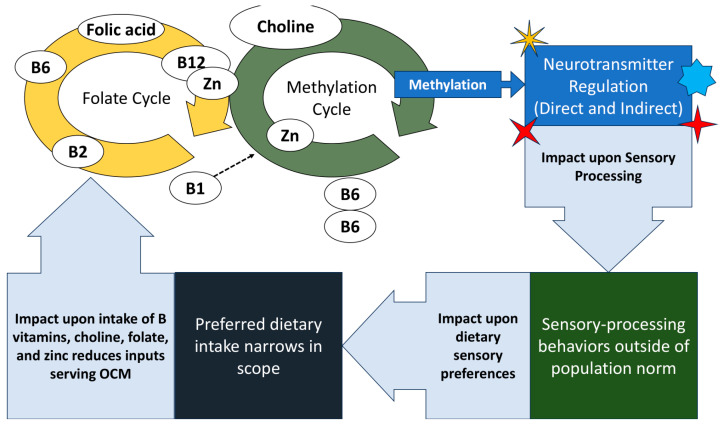
Potential feedback loop between OCM nutrient intake and sensory processing. This study’s data cannot rule out a mechanistic feedback loop in which dietary intake of OCM nutrients influences sensory processing, but in which sensory processing also influences the intake of OCM nutrients. Future research is necessary to clarify and define the nature of variable dependence.

Even so, this study has some notable limitations, including the sample size itself, which warrants mindfulness in the interpretation of these exploratory results. While the metabolism of OCM nutrients is interrelated (Figure 1), this study is not able to assess the implications of interactions of nutrients or varying levels of multiple nutrients. This study’s cross-sectional nature and small group size permits only identifying associations between the intake of any one OCM nutrient and oral sensory processing scores, and not which parties in that relationship serve as the dependent variable(s). Either one could serve as the dependent variable, or, in the case of a feedback loop, *both* can be dependent variables (Figure 4). Parent reports on both sensory aspects and dietary intake were a core aspect of this study’s data, which in each case was collected using a standardized, validated assessment. Though this study’s use of parent reports on the child’s diet through a food frequency questionnaire is a method which has been used successfully before in the literature, it remains somewhat subjective as a measure, to the extent that it depends heavily upon parent comprehension and interpretation of the assessments [82,83,84,85,86,87]. While all parents participating spoke English, English was not always the native language spoken by a parent, which could further cloud YAFFQ question interpretation—as it would any standardized dietary assessment method offered in English. Among other limitations, this study characterized food selectivity qualitatively with the judgment of the study’s two nutrition experts (MS and AO), without the use of a formal assessment tool to validate such characterization. The small sample size of this study did not permit greater diversity in SP2 category tallies.

## 5. Conclusions

To our knowledge, this is the first study to examine correlations between parent reporting data via the SP2 and YAFFQ assessment tools. In this group of school-aged children with ASD diagnoses, relationships were observed between the nutritional intake of choline, vitamins B1 and B12 intake, and certain SP2 sensory domain scores, supporting OCM nutrient intake in autistic people as a candidate topic for future related sensory processing research. The children in the study were observed to have a largely adequate nutritional consumption of most micronutrients, and sensory processing scores that were consistent with other studies of autistic children. This study’s identified associations serve future research with a profile of particular SP2 sensory processing domains of interest, as they relate to OCM nutrient consumption: Oral, Visual, Sensor, and Avoider. Given the cross-sectional nature of the data, it would be premature to interpret this study’s results as ready for clinical application. Future controlled trials would be needed to determine if modifying OCM intake would impact sensory symptoms in a meaningful way that would warrant changes in nutritional recommendations or clinical practice.

## Figures and Tables

**Table 1:** Participant characteristics (*n* = 33).

Variable	
Age (years)	8.0 ± 0.2
Age at ASD diagnosis (years)	3.8 ± 0.3
Gender	
Male	23 (69.7%)
Female	10 (30.3%)
Race/ethnicity	
White, non-Hispanic	19 (57.6%)
White, Hispanic	2 (6.1%)
Black, non-Hispanic	4 (12.1%)
Asian	4 (12.1%)
Biracial	4 (12.1%)
Types of therapy For ASD in the last 12 months	
Medication	10 (30.3%)
Occupational therapy	23 (69.7%)
Physical therapy	1 (3.0%)
Nutrition therapy/counseling	0 (0.0%)
Speech therapy	24 (72.7%)
Behavioral therapy	24 (72.7%)
Psychologist/Psychiatrist	7 (21.2%)
Social skills classes	9 (27.2%)
Music therapy	1 (3.0%)
Neurofeedback	1 (3.0%)
Medication use	
Use of any regular medication	20 (60.6%)
ADHD medication	15 (45.5%)
Antidepressant SSRI	4 (12.1%)
Bowel regularity	3 (9.1%)
Seasonal allergy	3 (9.1%)
Other	4 (12.1%)
Ever followed a specific diet for ASD	7 (21.2%)
Dietary supplements used currently	22 (66.7%)
Multivitamin/mineral	13 (39.4%)
Omega-3/fish oil	11 (33.3%)
Single vitamin or mineral	7 (21.2%)
Fiber	7 (21.2%)
Botanical	5 (15.2%)
Probiotic	3 (9.1%)
Other	3 (9.1%)
Child exhibits food selectivity	22 (66.7%)

**Table 2:** Intake of nutrients related to one-carbon metabolism in school-aged children with autism spectrum disorder (*n* = 33).

Nutrient	YAFFQ Intake (*n* = 33) (mean ± SD)	Number of Participants Meeting Suggested Intake Based on Age and Gender	% of Participants Meeting Suggested Intake Based on Age and Gender	Source for Intake Recommendation
Vitamin B1 (mg)	2.4 ± 1.9	33	100%	DRI (RDA)
Vitamin B2 (mg)	2.9 ± 2.0	32	97%	DRI (RDA)
Vitamin B6 (mg)	3.0 ± 2.1	32	97%	DRI (RDA)
Folate (mcg)	622.1 ± 362.5	30	91%	DRI (RDA)
Vitamin B12 (mcg)	8.3 ± 6.5	31	94%	DRI (RDA)
Choline (mg)	277.8 ± 112.7	12	36%	DRI (AI)
Zinc (mg)	16.2 ± 9.5	31	94%	DRI (RDA)

Abbreviations: AMDR = Acceptable Macronutrient Distribution Range; DGA = Dietary Guidelines for Americans 2020–2025; RDA = Recommended Dietary Allowance; DRI = Dietary Reference Intakes; AI = Adequate Intake.

**Table 3:** Child Sensory Profile 2 component scores of school-aged children with autism spectrum disorder (*n* = 33).

**Sensory Profile Quadrant Score**	**Mean (SD)**		**Median**	**Min**	**Max**
Seeking/Seeker	52.03	(16.31)	51	17	79
Avoiding/Avoider	56.33	(13.51)	56	25	89
Sensitivity/Sensor	51.36	(12.03)	53	26	74
Registration/Bystander	48.85	(17.64)	50	4	89
**Sensory Profile Factor Score**					
Auditory	23.91	(7.18)	24	8	38
Visual	14.91	(4.22)	15	4	24
Touch	24.30	(10.25)	24	3	41
Movement	18.55	(6.52)	19	3	36
Body Position	15.76	(8.08)	16	0	34
Oral	26.82	(10.07)	36	8	43
Conduct	24.03	(8.05)	24	10	41
Social Emotional	42.55	(10.54)	44	14	66
Attentional	27.91	(6.42)	27	16	42

**Table 4:** Tally distribution of Sensory Profile 2 categorization (*n* = 33). The normal distribution tally estimate gives a rough comparison of how the numbers might be divided, if instead 33 school-aged children were drawn randomly from the general population for an SP2 assessment. Grey colored cells occur where no participant from this study had a score classifying them in the given population distribution group.

	Much Less Than Others	Less Than Others	About the Same as Others	More Than Others	Much More Than Others
Seeker		1	13	8	11
Avoider			8	10	15
Sensor			6	11	16
Bystander	1	1	9	11	11
Auditory		1	17	10	5
Visual	1	1	23	6	2
Touch		2	10	8	13
Movement		2	14	13	4
Body Position	2	1	13	8	9
Oral			15	7	11
Conduct			14	11	8
Social			4	10	19
Attentional			10	12	11
	Greater than 2 standard deviations:	Between 1 and 2 standard deviations:	Within 1 standard deviation:	Between 1 and 2 standard deviations:	Greater than 2 standard deviations:
Theoretical distribution in general population out of 33, for comparison	0.44 0–1 person	4.49 4–5 people	22.44, 22–23 people	4.49, 4–5 people	0.44 0–1 person

**Table 5:** Relationships observed in linear regression model outcomes assessing median-binarized micronutrient intake with respect to Child Sensory Profile 2 component scores. Four out of the thirteen SP2 domains indicate relationships: the Avoider, Sensor, Visual, and Oral domains. (Results for all thirteen SP2 domains are presented in Supplementary Table S2). Bold font indicate *p*-values of less than 0.05.

	Avoider	Sensor	Visual	Oral
**Choline**				
*p*-val	**0.045**	0.195	0.070	0.503
beta	11.97	7.09	3.45	2.94
CI min, max	0.29, 23.65	−3.85, 18.03	−0.3, 7.2	−5.92, 11.8
**Vitamin B1**				
*p*-val	0.823	0.172	**0.026**	0.058
beta	1.13	−6.09	3.41	−6.6
CI min, max	−9.12, 11.38	−14.99, 2.81	0.44, 6.38	−13.43, 0.23
**Vitamin B2**				
*p*-val	0.768	0.202	0.052	0.075
beta	1.51	−5.78	3.05	−6.3
CI min, max	−8.88, 11.9	−14.85, 3.29	−0.03, 6.13	−13.28, 0.69
**Vitamin B6**				
*p*-val	0.779	0.208	0.671	0.095
beta	1.42	−5.66	0.68	−5.87
CI min, max	−8.87, 11.72	−14.65, 3.33	−2.58, 3.94	−12.84, 1.1
**Folate**				
*p*-val	0.609	0.139	0.616	0.085
beta	−2.57	−6.56	0.8	−6.01
CI min, max	−12.75, 7.61	−15.38, 2.27	−2.43, 4.03	−12.9, 0.88
**Vitamin B12**				
*p*-val	0.719	**0.047**	0.310	**0.026**
beta	−1.85	−8.89	1.64	−7.81
CI min, max	−12.3, 8.6	−17.64, −0.13	−1.61, 4.9	−14.62, −1
**Zinc**				
*p*-val	0.765	0.167	0.170	0.073
beta	−1.52	−6.2	2.17	−6.3
CI min, max	−11.82, 8.78	−15.14, 2.75	−0.99, 5.33	−13.21, 0.62

## Data Availability

The data presented in this study are available on request from the corresponding author.

## Supplementary Materials

The following supporting information can be downloaded at: https://www.mdpi.com/article/10.3390/nu17040604/s1, Supplementary Table S1: Nutrient Intake of School-aged Children with Autism Spectrum Disorder (*n* = 33), full nutrient data version. Supplementary Table S2: Linear regression model outcomes assessing median-binarized micronutrient intake with respect to all 13 Child Sensory Profile 2 component scores, version with full report from all 13 SP2 sensory domains. Supplementary Table S3: Linear regression model outcomes assessing deficient OCM nutrient intake with respect to raw sensory processing scores for oral, visual, and avoider sensory domains.
