# Supplementary material for: Nutritional Intake and Sensory Processing in School-Aged Children with Autism Spectrum Disorder
Source: Nutrients. 2025 Feb 7;17(4):604. doi: 10.3390/nu17040604 (PMC11858489; doi:10.3390/nu17040604)
Supplement: Supplementary file 1 [file nutrients-17-00604-s001.zip › nutrients-3368808-supplementary.pdf]

Supplementary Table S1. Nutrient Intake of School-aged Children with Autism Spectrum Disorder (*n*=33).

Nutrient	YAFFQ Intake (*n*=33) (mean ± SD)	Number of participants meeting suggested intake based on age and gender	% of participants meeting suggested intake based on age and gender	Source for Intake Recommendation
Kilocalories	1949.9 ± 594.7			
Total fat (g)	67.4 ± 24.8			
(% of calories)	30.7 ± 4.8	24	71%	AMDR
Saturated fat (g)	23.6 ± 9.7			
(% of calories)	10.6 ± 2.1	22	67%	DGA
PUFA (g)	15.2 ± 5.7			
MUFA (g)	22.9 ± 8.5			
Protein (g)	73.3 ± 28.5	32	97%	DRI (RDA)
(% of calories)	15.0 ± 3.8	31	94%	AMDR
Carbohydrate (g)	273.1 ± 87.8	33	100%	DRI (RDA)
(% of calories)	56.4 ± 6.8	29	88%	AMDR
Fiber (g)	21.6 ± 6.8			
(g/1,000 cal)	11.5 ± 3.3	8	32%	DGA
Sugar, total (g)	118.8 ± 46.8			
Added sugar (g)	58.2 ± 33.3	18	54%	DGA
Vitamin B1 (mg)	2.4 ± 1.9	33	100%	DRI (RDA)
Vitamin B2 (mg)	2.9 ± 2.0	32	97%	DRI (RDA)
Vitamin B3 (mg)	29.8 ± 14.9	32	97%	DRI (RDA)
Pantothenic acid (mg)	10.2 ± 7.4	30	91%	DRI (AI)
Vitamin B6 (mg)	3.0 ± 2.1	32	97%	DRI (RDA)
Folate (mcg)	622.1 ± 362.5	30	91%	DRI (RDA)
Vitamin B12 (mcg)	8.3 ± 6.5	31	94%	DRI (RDA)
Vitamin C (mg)	157.1 ± 127.1	33	100%	DRI (RDA)
Choline (mg)	277.8 ± 112.7	12	36%	DRI (AI)
Vitamin A (mcg RAE)	1174.9 ± 768.8	29	88%	DRI (RDA)
Vitamin D (IU)	610.1 ± 492.3	13	39%	DRI (RDA)
Vitamin E (mg)	17.4 ± 16.4	23	70%	DRI (RDA)
Vitamin K (mcg)	97.6 ± 66.5	26	79%	DRI (AI)
Potassium (mg)	2506.0 ± 802.2	18	54%	DRI (AI)
Sodium (mg)	2616.5 ± 868.0	32	97%	DRI (AI)
		4	12%	DRI (CDRR)
Calcium (mg)	1094.4 ± 457.3	17	52%	DRI (RDA)
Iron (mg)	21.4 ± 15.3	27	82%	DRI (RDA)
Zinc (mg)	16.2 ± 9.5	31	94%	DRI (RDA)
Magnesium (mg)	293.0 ± 79.0	31	94%	DRI (RDA)

Abbreviations: AMDR = Acceptable Macronutrient Distribution Range; DGA = Dietary Guidelines for Americans 2020-2025; RDA = Recommended Dietary Allowance; DRI = Dietary Reference Intakes; AI = Adequate Intake. Yellow cell shading indicates the one-carbon metabolism nutrients. Empty cells do not have recommendations defined.

Supplementary Table S2. Linear regression model outcomes assessing median-binarized micronutrient intake with respect to all 13 Child Sensory Profile 2 component scores.

	Seeker	Avoider	Sensor	Bystander	Auditory	Visual	Touch	Movement	Body Position	Oral	Conduct	Social	Attentional
**Choline**													
p-val	0.489	**0.045**	0.195	0.780	0.184	0.070	0.208	0.561	0.540	0.503	0.437	0.218	0.572
beta	5.22	11.97	7.09	-2.37	4.49	3.45	5.97	1.72	-2.33	2.94	2.99	5.83	-1.75
CI min, max	-10.03, 20.46	0.29, 23.65	-3.85, 18.03	-19.57, 14.84	-2.27, 11.24	-0.3, 7.2	-3.52, 15.45	-4.28, 7.73	-10.03, 5.37	-5.92, 11.8	-4.78, 10.76	-3.64, 15.3	-8.03, 4.52
**Vitamin B1**													
p-val	0.876	0.823	0.172	0.533	0.251	**0.026**	0.687	0.975	0.816	0.058	0.860	0.558	0.365
beta	-0.97	1.13	-6.09	-4.3	3.18	3.41	-1.58	-0.08	-0.72	-6.6	-0.56	-2.29	-2.28
CI min, max	-13.51, 11.58	-9.12, 11.38	-14.99, 2.81	-18.27, 9.66	-2.38, 8.74	0.44, 6.38	-9.53, 6.36	-5.01, 4.86	-7.04, 5.6	-13.43, 0.23	-6.97, 5.85	-10.18, 5.61	-7.36, 2.8
**Vitamin B2**													
p-val	0.620	0.768	0.202	0.279	0.375	0.052	0.568	0.600	0.713	0.075	0.372	0.913	0.133
beta	-3.1	1.51	-5.78	-7.53	2.51	3.05	-2.27	-1.29	-1.16	-6.3	-2.84	-0.44	-3.79
CI min, max	-15.78, 9.58	-8.88, 11.9	-14.85, 3.29	-21.5, 6.44	-3.19, 8.2	-0.03, 6.13	-10.31, 5.77	-6.27, 3.69	-7.57, 5.24	-13.28, 0.69	-9.25, 3.58	-8.5, 7.63	-8.81, 1.23
**Vitamin B6**													
p-val	0.563	0.779	0.208	0.748	0.499	0.671	0.837	0.809	0.542	0.095	0.766	0.990	0.880
beta	-3.58	1.42	-5.66	2.24	1.9	0.68	-0.81	-0.59	1.91	-5.87	-0.95	-0.05	0.39
CI min, max	-16.13, 8.96	-8.87, 11.72	-14.65, 3.33	-11.87, 16.35	-3.78, 7.57	-2.58, 3.94	-8.82, 7.2	-5.55, 4.36	-4.41, 8.22	-12.84, 1.1	-7.38, 5.49	-8.04, 7.94	-4.79, 5.57
**Folate**													
p-val	0.309	0.609	0.139	0.645	0.874	0.616	0.604	0.585	0.954	0.085	0.225	0.672	0.339
beta	-6.21	-2.57	-6.56	-3.17	0.44	0.8	-2.03	-1.32	-0.18	-6.01	-3.77	-1.65	-2.4
CI min, max	-18.5, 6.07	-12.75, 7.61	-15.38, 2.27	-17.15, 10.8	-5.23, 6.12	-2.43, 4.03	-9.94, 5.88	-6.22, 3.57	-6.49, 6.13	-12.9, 0.88	-10, 2.46	-9.55, 6.25	-7.46, 2.65
**Vitamin B12**													
p-val	0.378	0.719	**0.047**	0.812	0.750	0.310	0.541	0.504	0.712	**0.026**	0.743	0.471	0.688
beta	-5.53	-1.85	-8.89	-1.69	-0.91	1.64	-2.45	-1.65	-1.17	-7.81	-1.06	-2.87	-1.04
CI min, max	-18.17, 7.11	-12.3, 8.6	-17.64, -0.13	-16.04, 12.66	-6.72, 4.89	-1.61, 4.9	-10.53, 5.64	-6.65, 3.35	-7.62, 5.27	-14.62, -1	-7.59, 5.48	-10.91, 5.17	-6.29, 4.21
**Zinc**													
p-val	0.457	0.765	0.167	0.820	0.562	0.170	0.646	0.926	0.978	0.073	0.599	0.491	0.499
beta	-4.6	-1.52	-6.2	-1.58	1.63	2.17	-1.81	-0.23	-0.09	-6.3	-1.66	-2.7	-1.72
CI min, max	-17.1, 7.9	-11.82, 8.78	-15.14, 2.75	-15.72, 12.55	-4.06, 7.32	-0.99, 5.33	-9.79, 6.18	-5.19, 4.73	-6.45, 6.28	-13.21, 0.62	-8.08, 4.75	-10.62, 5.23	-6.86, 3.42

Supplementary Table S3. Linear regression model outcomes assessing deficient OCM nutrient intake with respect to raw sensory processing scores for oral, visual, and avoider sensory domains.

	**Choline Deficiency**	**B1 Deficiency**	**B2 Deficiency**	**B6 Deficiency**	**Folate Deficiency**	**B12 Deficiency**	**Zinc Deficiency**
**SP2 Avoider Domain Sensory Processing Raw Score**							
p-val	0.343	0.8996	0.901	0.901	0.5216	0.343	0.811
beta	0.0064	0	0.0003	0.0003	0.0026	0.0033	-0.0008
CI min, max	-0.0066, 0.0194	NA	-0.0046, 0.0052	-0.0046, 0.0052	-0.0103, 0.0052	-0.0034, 0.0100	-0.0074, 0.0058
**SP2 Visual Domain Sensory Processing Raw Score**							
p-val	0.093	0.974	0.878	0.877	0.428	0.284	0.807
beta	0.0352	0	0.0012	0.0012	-0.0100	0.0117	0.0026
CI min, max	-0.0045, 0.0749	NA	-0.0142, 0.0167	-0.0142, 0.0167	-0.0343, 0.0144	-0.0093, 0.0328	-0.0181, 0.0233
**SP2 Oral Domain Sensory Processing Raw Score**							
p-val	0.800	0.577	0.529	0.529	0.431	0.120	0.858
beta	0.0024	0	0.0022	0.0022	-0.0044	0.0075	0.0009
CI min, max	-0.0162, 0.0210	NA	-0.0046, 0.0091	-0.0046, 0.0091	-0.0153, 0.0064	-0.0017 0.0167	-0.0084, 0.0101
